# Accuracy and precision of consumer-level activity monitors for stroke detection during wheelchair propulsion and arm ergometry

**DOI:** 10.1371/journal.pone.0191556

**Published:** 2018-02-14

**Authors:** Jochen Kressler, Joshua Koeplin-Day, Benedikt Muendle, Brice Rosby, Elizabeth Santo, Antoinette Domingo

**Affiliations:** 1 School of Exercise and Nutritional Sciences, San Diego State University, San Diego, California, United States of America; 2 Institute of Human Movement Sciences and Sport, ETH Zurich, Zurich, Switzerland; 3 Doctor of Physical Therapy Program, San Diego State University, San Diego, California, United States of America; University of Illinois at Urbana-Champaign, UNITED STATES

## Abstract

The purpose of this study was to evaluate whether consumer-level activity trackers can estimate wheelchair strokes and arm ergometer revolutions. Thirty able-bodied participants wore three consumer-level activity trackers (Garmin VivoFit, FitBit Flex, and Jawbone UP24) on the wrist. Participants propelled a wheelchair at fixed frequencies (30, 45 and 60 strokes per minute (spm)) three minutes each and at pre-determined varied frequencies, (30–80 spm) for two minutes. Participants also freely wheeled through an obstacle course. 10 other participants performed arm-ergometry at 40, 60 and 80 revolutions per minute (rpm), for three minutes each. Mean percentage error (MPE(SD)) for 30 spm were ≥46(26)% for all monitors, and declined to 3-6(2–7)% at 60 spm. For the obstacle course, MPE ranged from 12-17(7–13)% for all trackers. For arm-ergometry, MPE was at 1-96(0–37)% with the best measurement for the Fitbit at 60 and 80 rpm, and the Garmin at 80rpm, with MPE = 1(0–1)%. The consumer-level wrist-worn activity trackers we tested have higher accuracy/precision at higher movement frequencies but perform poorly at lower frequencies.

## Introduction

Individuals with disabilities are twice as likely to be inactive compared to their healthy counterparts [1], leading to secondary complications such as obesity and cardiovascular disease [2]. Healthy People 2020, an evidence-based government program to improve the nation’s health, emphasises including people with disabilities in health promotion efforts [3]. Understanding disparities in health and physical activity (PA) between adults with and without disabilities is an integral part of this effort [1, 3]. It is therefore critical to develop tools to measure PA with accuracy and precision in people with disability. It would also important to test existing commercially available technologies to assess if they can be used for this purpose.

Consumer level PA monitors (PAM) present a convenient and cost-effective measurement of PA and can be used as motivation to increase PA [4]. PAM typically utilize a tri-axial accelerometer, which converts frequency and intensity data from user activity, to create a tally of steps as well as other functions [5]. PAMs can also be useful for clinicians to track and monitor a patient’s PA level. Several groups have studied the validity of these devices to measure steps during a variety of walking activities in able-bodied and clinical populations [5–21]. Generally, these studies showed that PAMs are a valid, low-cost method of measuring stepping, but are less accurate at slower walking speeds [12, 20, 21], depending on where the device was placed on the body [15, 16, 20].

Since many popular PAMs are worn on the wrist it is possible that they would capture movements other than swinging of the arms during walking such as wheelchair strokes. This would allow application of PAMs for wheelchair users. In a recent study, four out of five wheelchairs users indicated interest in using PAM but expressed concerns about accuracy/precision for wheel chair stroke counts [22]. The purpose of this study was therefore to evaluate the ability of consumer-level PAMs to accurately count arm strokes during activities common in everyday lives of wheelchair users (wheelchair propulsion and arm-crank ergometry). Based on previous research involving the use of PAM during walking, we hypothesized that wheeling at slower frequencies would result in less accurate counts of activity than at higher frequencies.

## Materials and methods

### Participants

30 able-bodied participants volunteered for the study (19 females; age (years): 23.8±3.9, height (cm): 167.6±8.7, and weight (kg): 68.7±16.5 [mean±SD]). For the arm ergometry task, ten different able-bodied participants were recruited (8 females, age (years): 25.4±5.8, height (cm): 165.4±8.8, and weight (kg): 64.1±10.5). The San Diego State University Institutional Review Board approved all procedures, and all participants provided written informed consent.

### Fitness trackers

Three wrist-worn commercially available PAMs (Garmin Vivofit, Fitbit Flex, and Jawbone UP24) were selected based on their popularity/affordability. All three PAMs have tri-axial accelerometers. Algorithms of these trackers are tuned for walking. Thresholds are set to determine a large enough movement indicative of walking while also attempting to minimize counting of smaller movements not associated with stepping. Based on our empirical observations of the relationship between PAM counts and wheelchair strokes, one stroke on the wheelchair was logged as two counts on the PAM. For the arm ergometry tasks, we counted one revolution as one count on the fitness trackers.

### Protocol

#### Wheelchair tasks

Participants propelled a wheelchair while wearing all activity trackers on their right wrist, placed in random order. Participants were given time to practice before collecting data to avoid a learning effect. They were also given breaks between each bout of wheeling.

**Fig 1:**
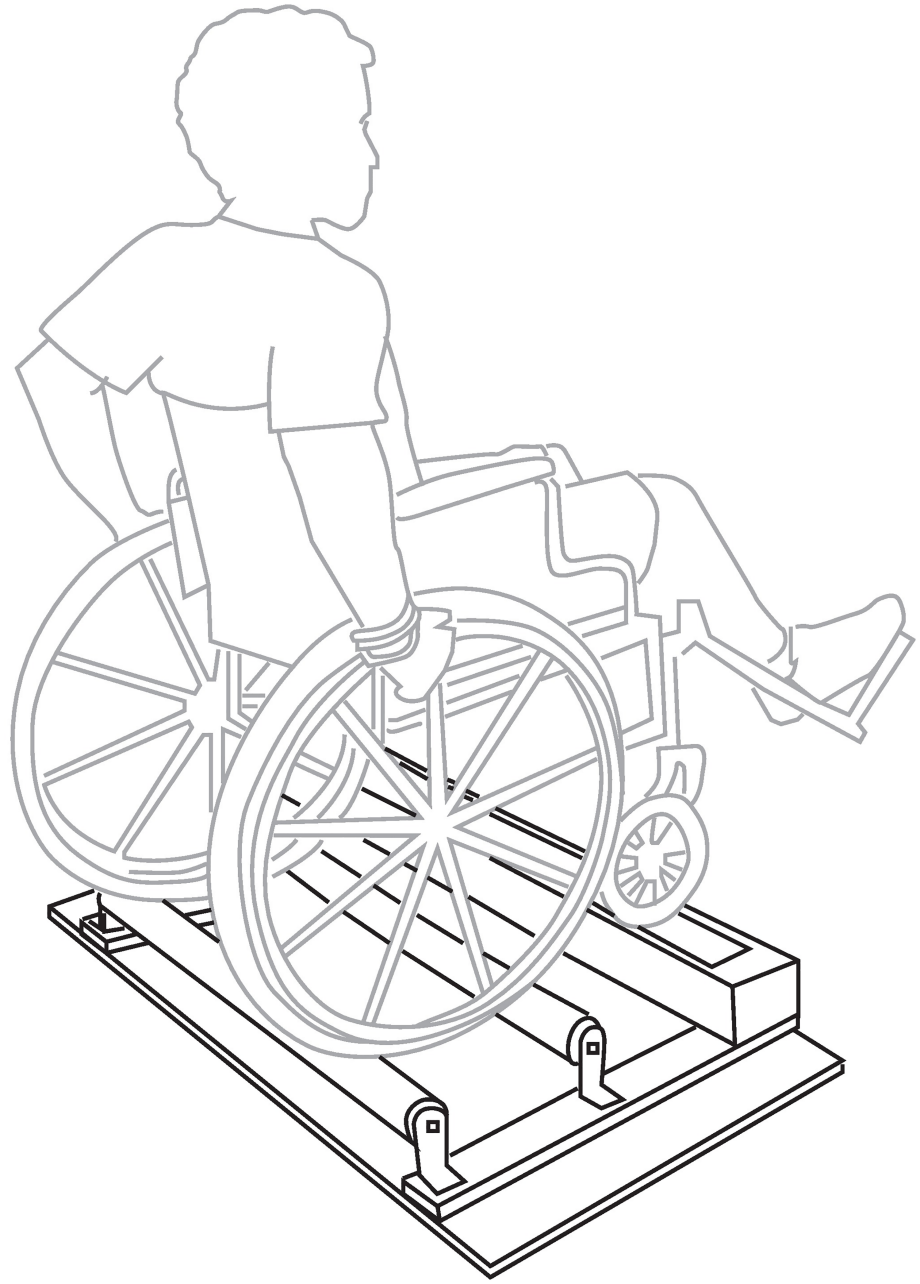
Wheelchair rollers setup. An illustration of the participant propelling the wheelchair on suspended rollers.

**Fig 2:**
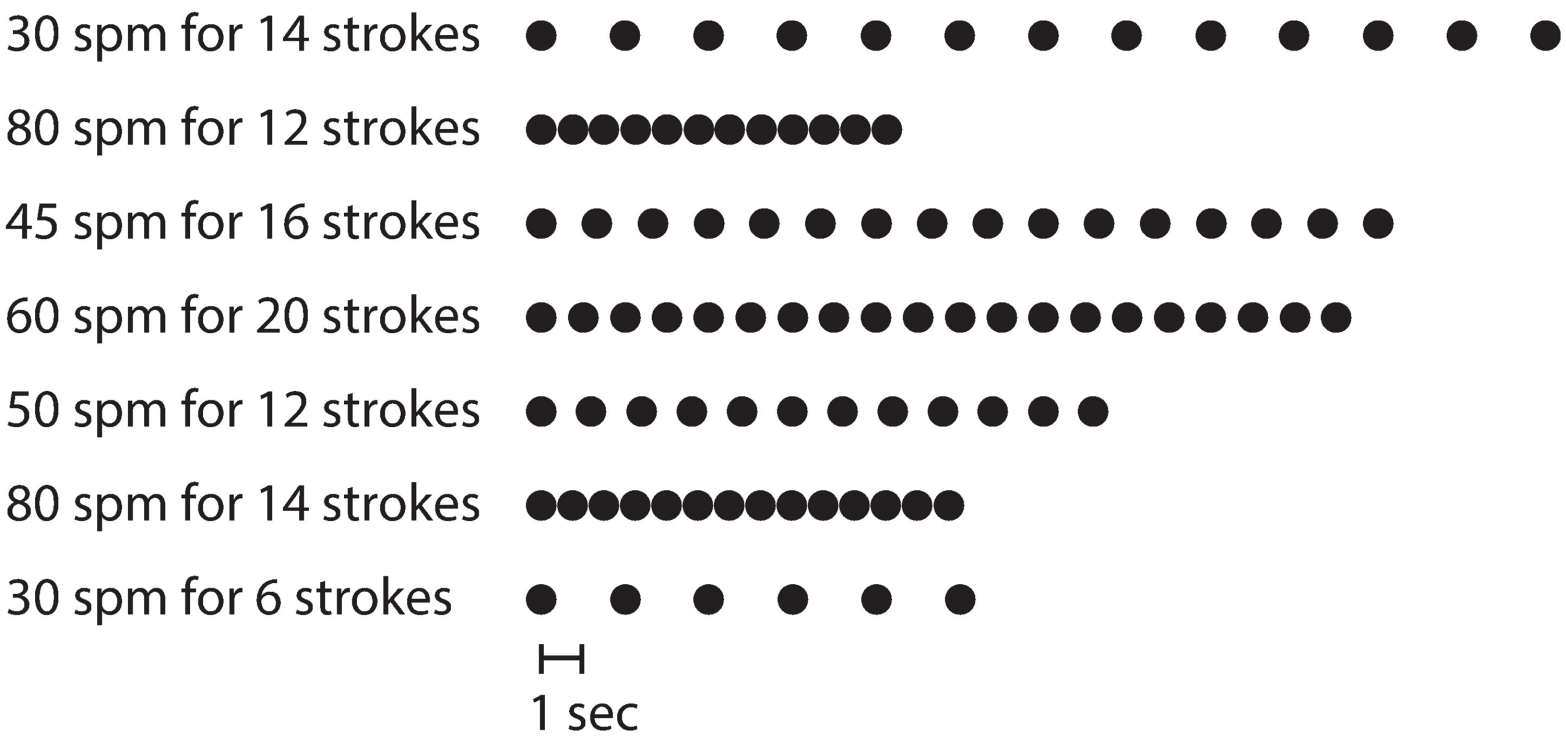
Mixed frequencies trial. A graphic representation of the frequencies and number of strokes performed for the Mixed condition during the rollers task.

For the rollers task, participants propelled the wheelchair on suspended rollers (Fig 1) at separate frequencies (30, 45 and 60 strokes per minute (spm)), three minutes each. These frequencies were selected based on a previous study where experienced wheelchair users propelled at self-selected speeds over ground at an average of ~53 spm [23]. Participants also propelled the wheelchair on the rollers at pre-determined varied frequencies (Mixed), ranging from 30–80 spm over 2 minutes (mean = 47 spm, Fig 2). Each frequency was performed 3 times and averaged before statistical analysis was performed, except for within-tracker comparisons. We used a metronome to allow participants to easily adhere to the prescribed frequencies, and gave them the opportunity to practice the frequencies before starting the data recordings. Participants were visually monitored throughout the trial and given verbal cues as needed to ensure they followed the metronome. The order of trials was randomized between each participant.

**Fig 3:**
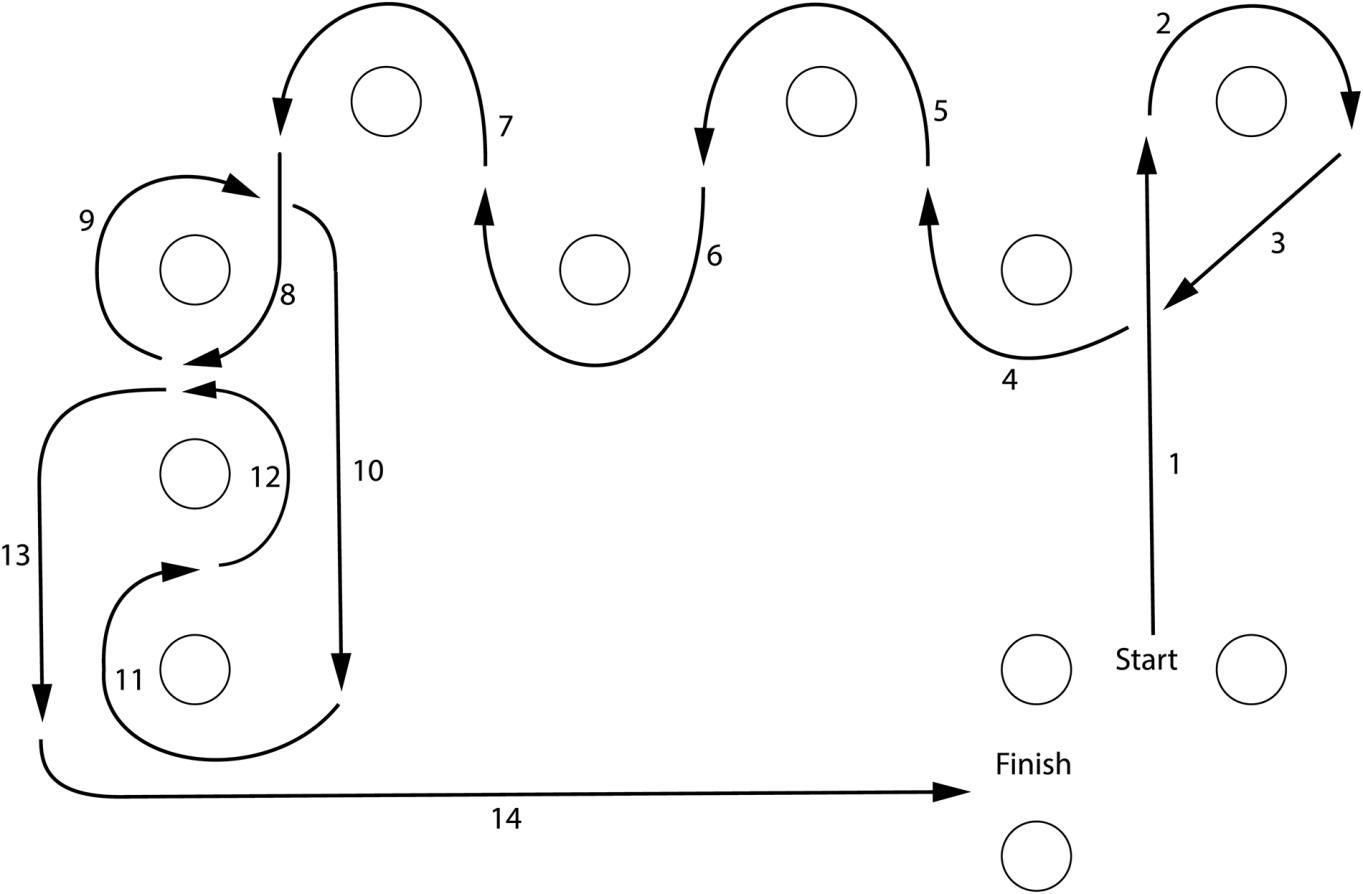
Obstacle course depiction. The obstacle course negotiated by participants in a manual wheelchair. The course covered ~9m x7.5m area.

Participants also wheeled through an obstacle course twice (Fig 3) at self-selected speeds. Two experimenters used tally counters to count strokes of participants’ right arm. If there was a non-zero difference between the observers, the trial was repeated. For a subset of participants (*n* = 19), time to complete the obstacle course was recorded to calculate mean spm.

#### Arm ergometry

A second group of participants performed an arm ergometry task. The arm ergometer (Drive Medical, Model RTL10273) was placed at a participant-selected height and distance. Participants cycled at different frequencies (40, 60 and 80 revolutions per minute (rpm)) for three minutes each. This range of frequencies was selected based on those used previously involving arm ergometry and hand cycling in wheelchair users [24–26]. The number of revolutions performed was verified using the display count on the ergometer.

### Data analysis and statistics

Data were analysed with Statistical Packaging for Social Sciences 22 (SPSS, IBM, Armonk, NY) unless indicated otherwise. Data are presented as mean across trials (95% confidence intervals lower bound, upper bound, unless indicated otherwise) for stroke counts, and mean percentage error (MPE) (Table 1). Standard Error of Measurement (SEM) was calculated for combined systematic and random error as the square root of within subject mean squares as described by Weir et al. [27].

**Table 1:** Standard equations for descriptives.

	Equation	#
Mean Count	$\frac{\sum Ci}{k}$	1
MPE	$\sum \frac{\left|Ci-Ti\right|}{Ti}*k^{-1}*100$	2

C = count by tracker; i = trial; k = number of trials; T = adjusted pre-determined (rollers) or true (obstacle course and ergometer) stroke count.

For the rollers and arm ergometry tasks, a mixed design Analysis of Variance (ANOVA) with repeated measures was used to compare tracker counts and pre-determined (rollers) or true (arm ergometry) values across stroke frequencies. A separate ANOVA was also performed for MPE to compare each tracker error to zero across stroke frequencies, as well as comparing MPE between trackers.

For the obstacle course task, a one-way ANOVA with repeated measures was used to assess differences across tracker counts and the true values. An ANOVA was also performed for MPE to compare each tracker error to zero across stroke frequencies, as well as comparing MPE between trackers.

For all ANOVA analysis, if sphericity did not hold (or was undefined) the Huynh-Feldt adjustment was used to evaluate main effects of the within subjects variable and/or the interaction effect. Post-hoc analyses were done without adjustment for multiple comparisons. Effect sizes for main effects or interactions are presented as ratio of effect variability to error variability (partial eta squared, *η*_*p*_^*2*^) and for regressions as effect variability to total variability (*R*^2^).

For the obstacle course task, intra-class correlation coefficients (ICC) with 95% CI were calculated using a 1-way random model for average measures[27]. Lin’s concordance coefficients with 95% CI were calculated using open online statistical software [28]. ICC and Lin’s coefficient were not calculated for trials with predetermined outcomes values (i.e., the rollers and ergometer tasks) because there was no variability between participants in these tasks.

For the obstacle course task, modified Bland-Altman plots were created to plot difference values against observed (true) values. In addition to standard limits of agreement (LoA), we plotted minimal clinically important difference (MCID) [29], based on interval size for categorizing PA levels relative to target PA recommendations [30] at 25% of true values and centered on 0. Consistent error as mean difference scores (across all values) was assessed for significant difference from zero via single measurement *t*-test. Proportional bias was assessed with simple linear regression of difference values on observed values.

Within tracker reliability was assessed with a two-way random model with absolute agreement type [27] for each tracker across all 3 trials at each frequency for the roller and ergometer tasks. Heuristics for interpretation are based on Koo and Li [31], and are as follows: ICC values of less than 0.50 indicates poor reliability, ICC values in the range 0.50 to 0.75 indicate moderate reliability, between 0.75 and 0.9 indicates good reliability, and an ICC value of greater than 0.9 shows excellent reliability.

Level of significance was set at *α*≤.05.

## Results

### Wheelchair rollers tasks

**Fig 4:**
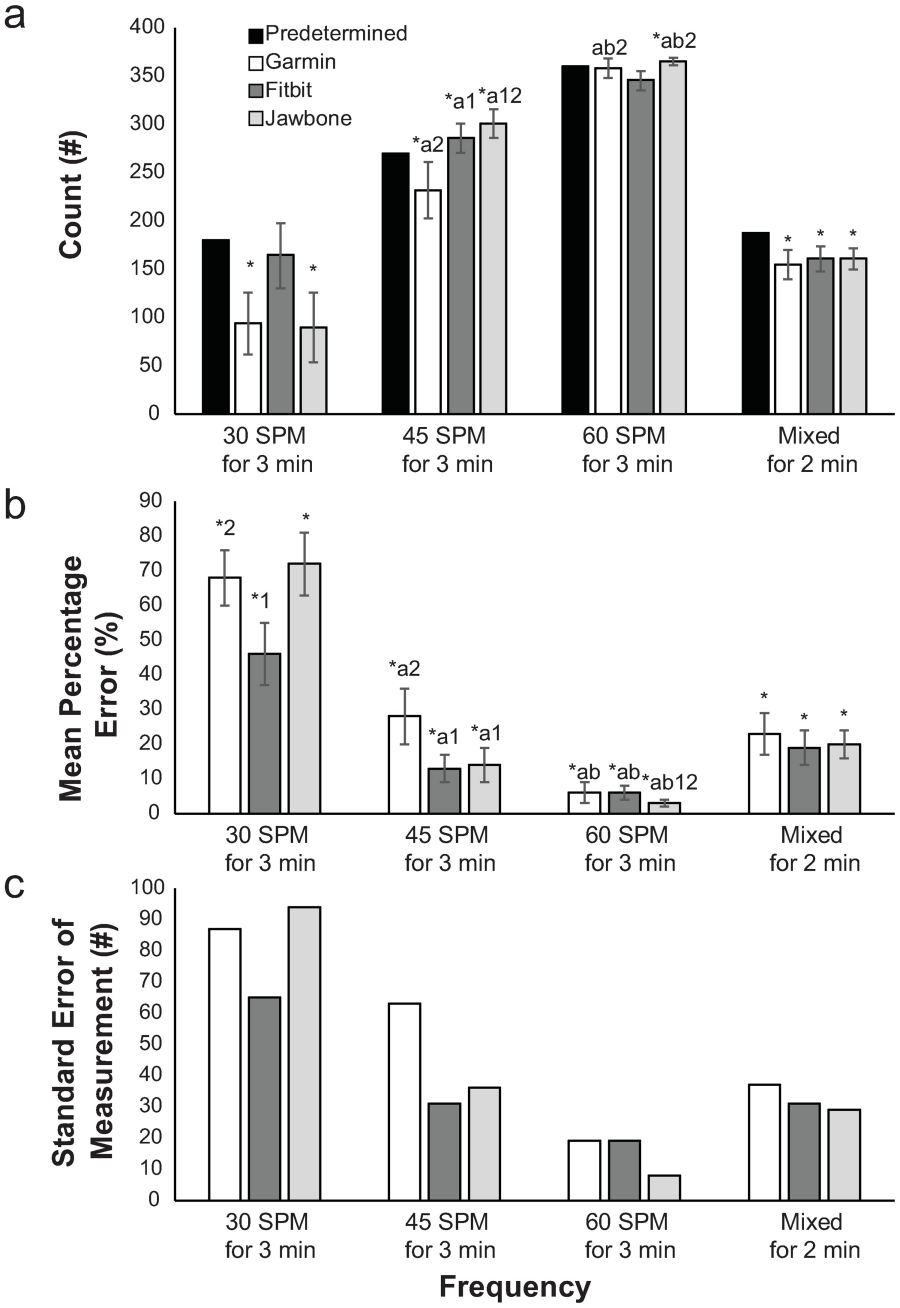
Wheelchair rollers task statistics. Means and 95%CI for (a) counts, (b) mean percentage error, and (c) standard error of measurement. Significant difference (*p*≤.05) *from Predetermined, ^a^from 30 spm, ^b^from 45 spm, ^1^from Garmin, ^2^from Fitbit.

For the rollers task, there was a significant interaction among tracker and pre-determined values across different speeds (*p* < .001, *η*_*p*_^*2*^ = .385) for mean counts. For individual trackers, mean counts showed significant differences for tracker-measured values compared to pre-determined values for most of the frequencies except for the Fitbit at 30 spm and the Garmin at 60 spm (Fig 4a).

In general, the trackers had smaller percent errors at the higher stroke frequencies. For all trackers, MPE significantly decreased (all *p* < .001, *η*_*p*_^*2*^≥.694) with increasing stroke frequency. Consistent with this, the SEM values were lower at higher stroke frequencies (Fig 4c). At 30 spm, SEM values for all trackers were high, but the Fitbit was the lowest. At 45 spm, the FitBit and Jawbone were closer to the true values (lower SEM), and at 60 spm, SEM was the lowest, and the Jawbone was closest to the pre-determined value.

During the mixed frequencies rollers trials, the counts for all trackers were significantly lower than the pre-determined counts (all Δ = -33(-48,-17) to -27(-40,-14), *p* < .001). When comparing the MPE for each tracker to each other, there were no significant differences (*p*≤.120, *η*_*p*_^*2*^≥.070, Fig 4b). SEM for each of the trackers were about the same, with the Garmin having slightly higher SEM than then the others.

Within tracker reliability for the rollers task was poor to moderate except for the Fitbit at 30 spm, and the Jawbone at 30 spm (Table 2).

**Table 2:** Within tracker reliability for each frequency.

		Garmin		FitBit		Jawbone	
		*ICC* (95% *CI*)	*p*-value	*ICC* (95% *CI*)	*p*-value	*ICC* (95% *CI*)	*p*-value
Rollers	30spm	0.697 (0.525–0.829)	< .001	0.818 (0.697–0.902)	< .001	0.780 (0.610–0.879)	< .001
	45spm	0.580 (0.376–0.752)	< .001	0.692 (0.517–0.825)	< .001	0.605 (0.406–0.769)	< .001
	60spm	0.156 (-0.053–0.406)	.077	0.627 (0.433–0.784)	< .001	0.255 (0.034–0.498)	.011
	Random	0.477 (0.257–0.679)	< .001	0.640 (0.450–0.792)	< .001	0.535 (0.322–0.721)	< .001
Ergometer	40rpm	0.258 (-0.112–0.686)	.094	0.265 (-0.106–0.691)	.088	0.205 (-0.151–0.650)	.144
	60rpm	0.499 (0.107–0.821)	.006	0.373 (-0.017–0.756)	.031	0.187 (-0.164–0.637)	.164
	80rpm	-0.001 (-0.282–0.473)	.477	-0.078 (-0.323–0.388)	.634	0.438 (0.044–0.791)	.014

### Obstacle course task

**Fig 5:**
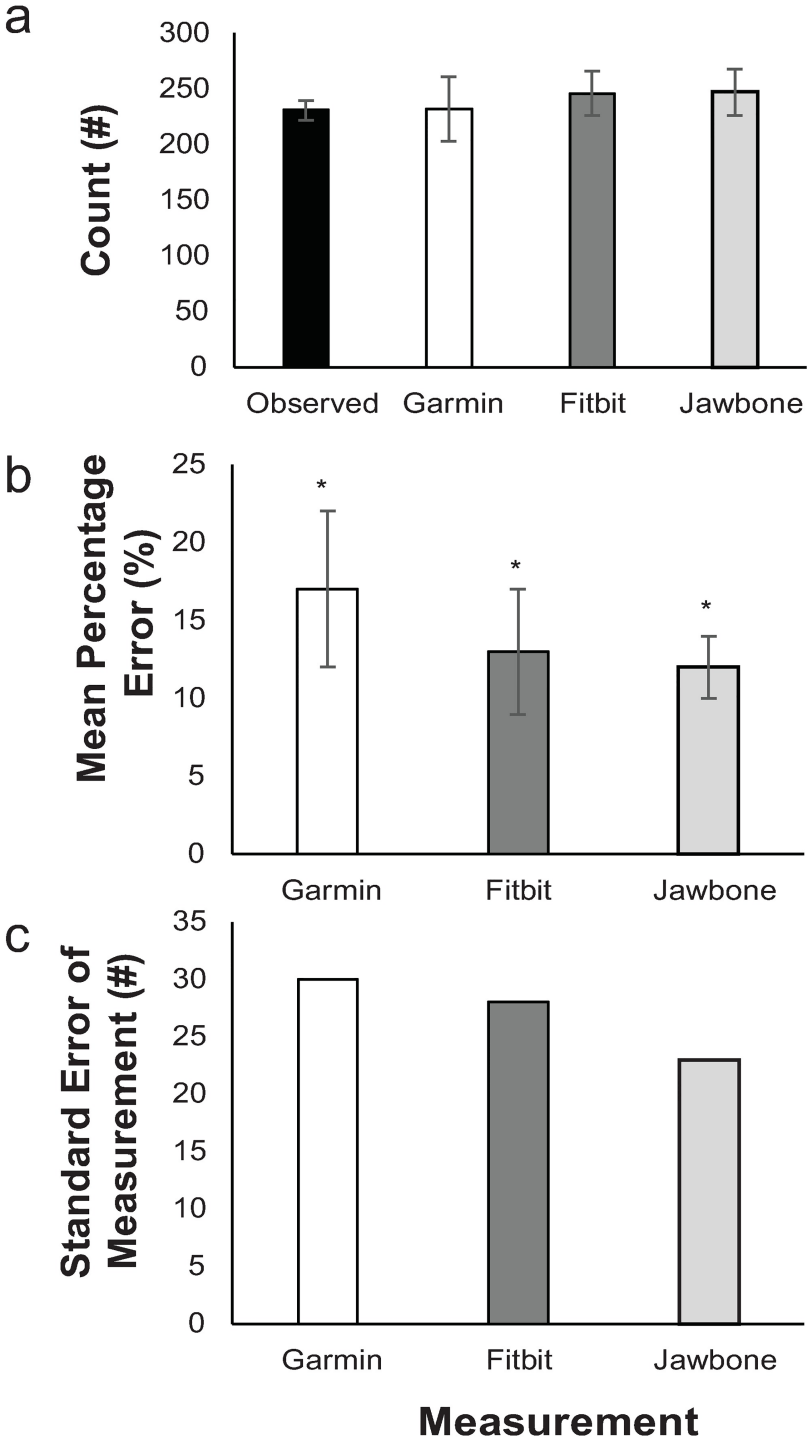
Obstacle course task statistics. Means and 95%CI for (a) counts, (b) mean percentage error, and (c) standard error of measurement. *significant difference from true (p≤.05).

For the obstacle course task, stroke frequency through the obstacle course was 48(44, 53) strokes per minute. The overall ANOVA showed no significant difference (*p* = .102, *η*_*p*_^*2*^≥.076) for the counts measured by trackers compared to true stroke count (Fig 5a). There was an overall effect of trackers on MPE (*p* < .001, *η*_*p*_^*2*^≥.469). Post-hoc analysis showed that MPE for all trackers were significantly different from zero (i.e. observed MPE, all<-12(-21,-9), *p* < .001, Fig 5b). There were no significant differences in MPE among the devices (*p≥*.138, *η*_*p*_^*2*^≤.066).

**Fig 6:**
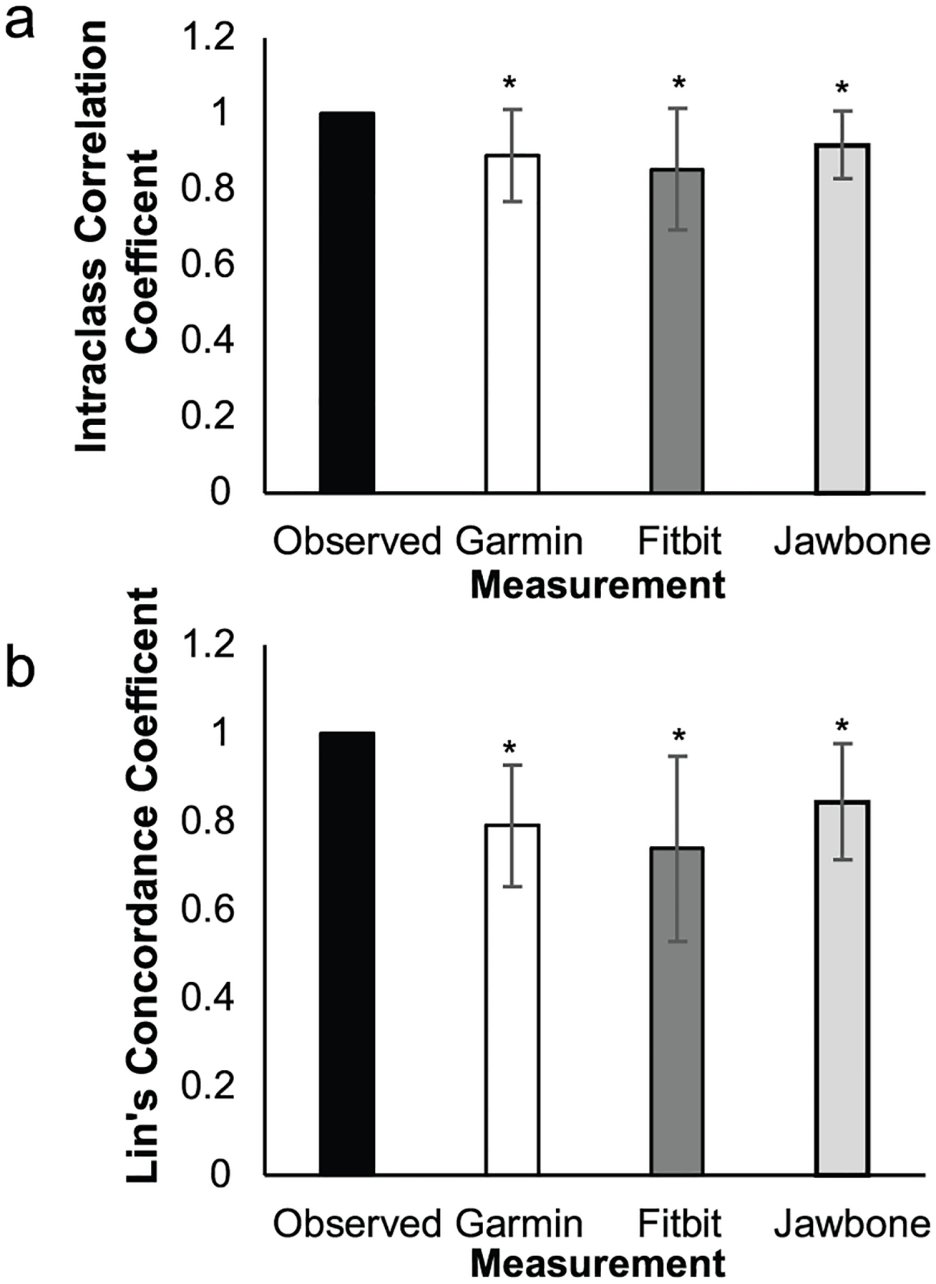
Statistic and 95%CI for obstacle course task. *Significant difference from 0 (p≤.05).

The ICC and Lin’s concordance coefficient were similar (as indicated by overlapping confidence intervals) between trackers, with values about 0.03 and 0.05 higher for Jawbone compared to Garmin (ICC and Lin’s coefficient, respectively) (Fig 6a and 6b), and the Garmin was about 0.04 and 0.05 higher than Fitbit.

**Fig 7:**
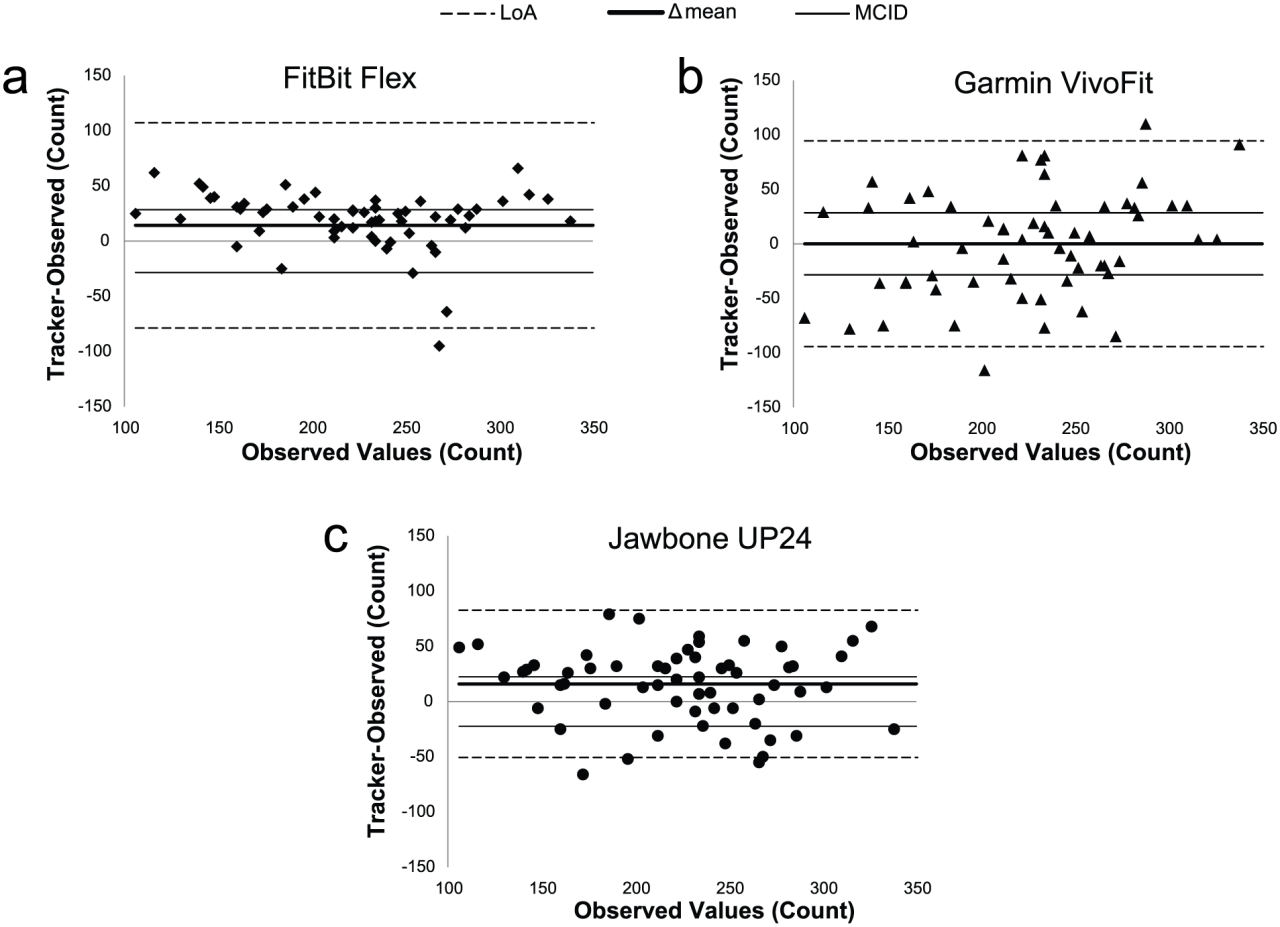
Modified Bland-Altman plots for obstacle course task. Observed values on x-axis. LoA were centered on 0. MCID = 25% of observed values. LoA, Limit of Agreement; MCID, minimal clinically important difference.

Bland-Altman plots (Fig 7a–7c) showed LoAs well above the MCID for all trackers. Only 45, 43 and 53% of difference values fell within the MCID for Garmin, FitBit and Jawbone, respectively. Consistent error was not significant for the Garmin (Δ_error_ = -0.1(-15,16), *p* = .990) but significant for the Fitbit (Δ = 14(1,28), *p* = .024) and the Jawbone (Δ = 16(6,26), *p* = .004). Proportional bias was not significant for any of the trackers (*p≥*.0.84, *R*^*2*^≤.103).

### Ergometer task

**Fig 8:**
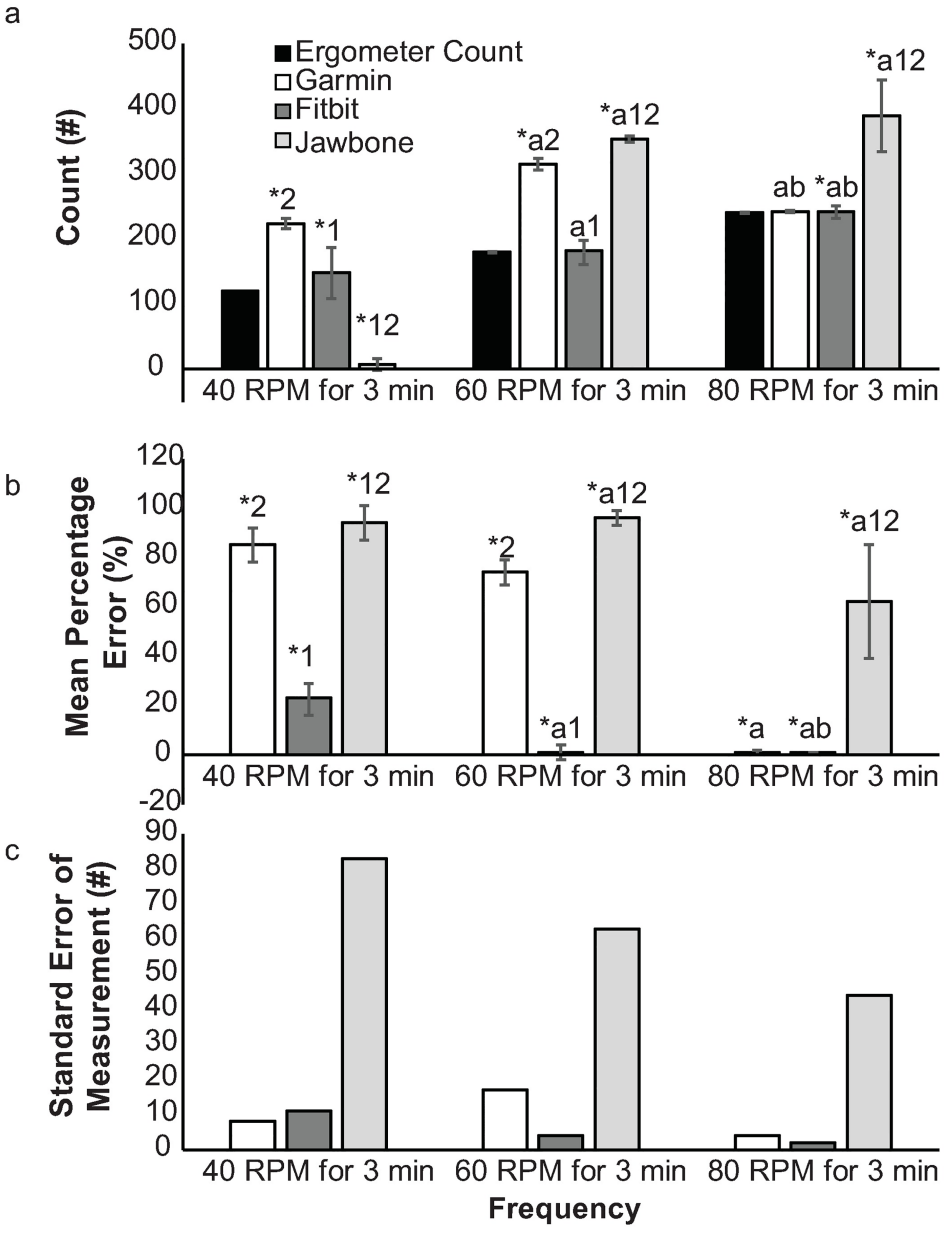
Ergometer task statistics. Means and 95%CI for (a) counts, (b) mean percentage error, and (c) standard error of measurement. Significant difference (p≤.05) *from true values, ^a^from 40 rpm, ^b^from 60 rpm, ^1^from Garmin, ^2^from Fitbit.

For the ergometer task, there was a significant interaction for tracker and frequency (*p* < .001, *η*_*p*_^*2*^≥.924). All trackers at all stroke frequencies had significantly different mean counts from the true counts (all *p* < .001, mean difference = -113 to 173, Fig 8a). For 23 of 30 trials at 40 rpm, the Jawbone recorded 0 counts. MPE for each trackers were significantly different than zero at each frequency (all MPE≥1(0–101), *p*≤.003, Fig 8b). The differences in MPE among cycle frequencies varied by tracker (*p* = .001, *η*_*p*_^*2*^≥.647). For the Garmin, all frequencies were significantly different than each other, except for between 40 and 60 rpm (Δ = 12(-1, 24), *p* = .066). The errors were less with higher speeds for the Garmin. For the FitBit, all errors were significantly different between the different frequencies, with higher errors 40 rpm, except there were virtually no errors at 60 and 80 Hz (Δ = 0(0,1), *p* = .317). For the Jawbone, the only significant differences among errors across all three frequencies was between 60 and 80 rpm (Δ = 34(3, 65), *p* = .034), as the MPE only decreased at the highest frequency.

Within tracker reliability for the ergometer tasks was poor to moderate for all trackers at all frequencies (Table 2).

## Discussion

The aim of this paper was to evaluate the ability of three popular consumer-level PAMs to detect strokes during different tasks: propelling a wheelchair at different frequencies, negotiating an obstacle course and using an arm ergometer. These fitness trackers exhibited poor accuracy and precision in measuring true strokes across a range of wheelchair tasks at lower to medium movement frequencies, but performed better at the higher frequencies we tested. Based on the ICC calculations, within PAM reliability was poor to moderate in almost all conditions. During the wheelchair rollers tasks, the three trackers were better at counting strokes at the highest frequency we tested (3–6% MPE), with no significant difference among trackers. The devices tested had substantial error during wheeling and low frequency arm ergometry (as demonstrated by high MPE and SEM). During the obstacle course task, errors for the trackers beyond the MCID occurred in about half of the trials. Only arm ergometry at the highest frequency tested (80 rpm) was measured with high accuracy and precision by two of the three trackers (Garmin and FitBit). Generally, the existing software algorithms to measure steps in these trackers are poorly suited to measure many common modes of arm exercises.

The PAMs tested in the current study tended to underestimate stroke counts on the rollers and overestimate obstacle course counts and ergometer revolutions. The MPEs at the higher movement frequencies we tested during wheelchair propulsion are similar to those from other studies using wrist-worn PAMs during walking or jogging [5, 7, 11].

For the Garmin and Jawbone during the rollers condition at 30 spm, it may appear that the method of doubling the actual stroke counts to match those of the PAM counts would be incorrect. There is a possibility that people pushed at a higher acceleration than what would be needed at 30 spm and then returned arms back to the starting position at a lower acceleration to stay on rhythm, and therefore only the forward stroke was counted. However, the variability is very large, 95% CI ranges = 64 and 72, respectively, for a 90 stroke count. Therefore the overall conclusion of poor validity and reliability for the trackers at low frequency seems defensible even if the stroke counts were not doubled.

There has been increasing attention on the use of low cost, consumer-level sensors to promote PA by changing exercise behavior. Bravata and colleagues [4] showed that having a simple pedometer can increase PA and others report that PAM have the potential to stimulate behavior change to potentially improve fitness and health [32–34]. Similar to able-bodied individuals, many wheelchair users have a desire to track their personal PA with a wearable device, as well as compare their own activity to family and friends using this device [22]. Based on the results of this study, the monitors we tested are not readily able to comprehensively measure activities of manual wheelchair users. Recently, Apple, Inc. has released a software update for the Apple Watch targeted for wheelchair users, allowing them the same access to the tools and social platforms available to able-bodied people to track their PA. However, the cost of this device might be prohibitive for many individuals (≥$299.00), and a lower cost consumer-level device (similar to the ones tested in this study) would enable more wheelchair users to monitor their PA and improve their health.

One limitation of this study is that we tested able bodied participants rather than experienced wheelchair users. A previous study showed that experienced manual wheelchair users have different frontal plane shoulder movements than novice wheelchair users during level wheelchair propulsion [35]. However, it is unlikely that the trackers we tested in this study would be sensitive enough to detect differences between experienced and novice wheelchair users. In addition, for the arm ergometry task, the movement is so highly constrained that it would be very unlikely for the trackers to discern between able-bodied and disabled participants.

Another limitation is the fact that we only measured short bouts of activity, and it is possible that longer bouts may influence the PAM measurement error. However, we would consider that most bouts of activity are likely short in duration (e.g., moving from desk to bathroom, wheeling around the house, getting from the car to a restaurant or store, etc). Although it is common to measure accuracy and reliability during short bouts of activity [12, 20, 36], it would be important to test the accuracy of these devices in ecologically valid settings in future studies.

In conclusion, our study showed that the consumer-level wrist-worn activity trackers we tested performed poorly in measuring arm strokes at lower to medium frequencies during wheelchair propulsion and arm ergometry, but performed better at higher frequencies. These trackers are therefore unlikely to accurately and precisely measure overall activity for most wheelchair users, highlighting the need for software that is specifically designed to measure activities commonly performed by persons with lower limb paralysis and weakness.

## Supporting information

S1 Dataset Study data. (XLSX)
